# Lack of knowledge of thalassemia among non-medical university students in Saudi Arabia: a cross-sectional study

**DOI:** 10.3389/fpubh.2026.1722773

**Published:** 2026-03-04

**Authors:** Noara Alhusseini, Peter M. B. Cahusac, Yara O. Alsouss, Sabri Kemahli

**Affiliations:** College of Medicine, Alfaisal University, Riyadh, Saudi Arabia

**Keywords:** awareness, health education, knowledge, Saudi Arabia, thalassemia, university students

## Abstract

**Background:**

Thalassemia is a prevalent hereditary hematological condition in Saudi Arabia, with carrier frequencies between 1.8 and 3.2%. Despite the implementation of the national premarital screening program in 2004, public awareness continues to be minimal. University students, anticipated to exhibit elevated health literacy, may still lack adequate knowledge.

**Methods:**

A cross-sectional study employed an online questionnaire distributed to undergraduate and graduate students from non-medical colleges at Alfaisal University. A17-item online survey assessed knowledge of thalassemia. The authors classified the knowledge scores into four ordinal categories and used ordinal regression to examine their associations with demographic factors.

**Results:**

Among the 223 responders, 69.5% had never heard of thalassemia. Approximately 48% scored zero on the knowledge testing, while 8% exhibited good knowledge. Nationality emerged as the only significant predictor of high knowledge (OR = 1.975, *p* < 0.01), with non-Saudi students demonstrating greater awareness. No associations were found with age, gender, or marital status.

**Conclusion:**

The results indicate an essential gap in thalassemia awareness, especially among educated young individuals. This gap and comprehensive community-based educational initiatives should be targeted at the middle and high school levels. Specific measures are essential to enhance national screening and preventive strategies. These findings highlight the urgent need for national educational initiatives to complement premarital screening, targeting younger populations before reproductive age.

## Introduction

Hemoglobinopathies, including thalassemia and sickle cell disease, are among the most prevalent genetic disorders worldwide (1). In Saudi Arabia, the burden is especially significant due to increased rates of consanguineous marriages, affecting about 50 % of the population (1).

National data indicate that the prevalence of *β*-thalassemia carriers varies between 1.8 and 3.2%, with the greatest rates seen in the Eastern and Southwestern areas (1). Consequently, the Saudi Ministry of Health initiated the Premarital Screening and Genetic Counseling (PMSGC) program in 2004 (1). This compulsory program seeks to reduce the prevalence of hemoglobinopathies by screening all couples before marriage and providing genetic counseling based on the findings (1). The program helped in reducing the overall prevalence. During a 5-year period the prevalence rate for *β*-thalassemia trait was found to decrease from 24.2 per 1,000 population in 2011, to 12 in 2015, as reported by the analysis of national data (2). Despite these efforts, several studies indicate that public understanding and awareness of thalassemia remain insufficient. Similar studies conducted in other countries with a high prevalence of thalassemia carriers gave similar results (3–7).

When examples of education programs on thalassemia and hemoglobinopathies are examined, the positive effects of the training are seen in different countries such as Indonesia, Turkey, Malaysia and Cambodia (8–13).

Families of patients with thalassemia were also included in the knowledge level assessments in some studies. A study in Indonesia reported that parents have a good knowledge of thalassemia, which increased even more with online education (4). An important point is the necessity of monitoring and follow-up after educating the target audience (11). A few studies are reported regarding the knowledge and awareness of the population about the PMS program in Saudi Arabia. In a study conducted among a randomly selected national sample of the Saudi population, all 893 participants had heard about PMS and 70% had satisfactory knowledge about the program (14). Another study performed among 1,047 individuals showed a “fair knowledge” about the PMS program, the tests and the targeted disorders (15). A study performed among college students in Bangladesh revealed that 67% of the students had never heard about thalassemia (5). An interventional study aimed to assess and improve the awareness of university students in Saudi Arabia revealed significant changes in knowledge and attitude of students about hemoglobinopathies as well as the need for accessible and continuous health education programs (16). Although PMSGC awareness has been studied, comprehensive thalassemia literacy (including inheritance, carrier status, symptoms, and misconceptions) among non-medical university students in KSA is still little documented. Considering the lack of sufficient data for Saudi Arabia, this study was planned to determine the knowledge level of non-medical university students about thalassemia.

## Methods

### Study design and setting

This cross-sectional study was conducted at Alfaisal University in Riyadh. It utilized a convenience sample method to recruit participants from the student population.

Ethical Consideration

This study was carried out in compliance with ethical standards for human research. No identifying data was collected. Data was stored securely and analyzed using appropriate statistical software.

Participants’ confidentiality and privacy were maintained throughout the study. Informed consent was obtained electronically before participation in the survey. The Institutional Review Board IRB approval was obtained from Alfaisal University before data collection (No. IRB20293).

The authors declare no conflict of interest.

### Participants

Participants were recruited exclusively from Alfaisal University (Riyadh). Eligible participants included current undergraduate and graduate students from all colleges of Alfaisal University, excluding those from the College of Medicine. The authors excluded medical students due to their prior exposure to thalassemia-related knowledge in the curriculum, which could bias the knowledge assessment.

### Data collection

Data were collected using an anonymous, self-administered online questionnaire distributed exclusively among Alfaisal University students using Google Forms. We distributed the survey link through official student email channels and social media, including WhatsApp.

### Survey instrument

The survey had two segments—the initial component collected demographic data, encompassing age, gender, nationality, and college affiliation. The second segment had 17 factual statements regarding thalassemia. Participants selected one of three response options for each item: “Yes,” “No,” or “I don’t know.” Correct responses were awarded a score of 1, but incorrect answers or those reflecting uncertainty earned a 0. The cumulative knowledge score ranged from 0 to 17. The scores were classified as no knowledge (no correct answers), poor (1–7 correct answers; 5–42%), medium (8–11 correct answers; 47–65%) and good (12–17 correct answers; 70–100%). The constructed survey was evaluated and approved by two field experts to ensure content validity. The survey was in English.

### Sample size

The data were collected from an overall student population of 3,000 students. Using a 95% confidence level and a 5% margin of error, there were a total of 223 respondents who participated on a voluntary basis.

### Statistical analysis

Data were analyzed utilizing jamovi (version 2.6 jamovi project).^1^ Descriptive statistics were applied to represent demographic characteristics and knowledge scores. Welch’s *t*-test and likelihood ratio (*G*) tests were used. Inferential analyses, encompassing correlation and ordinal regression, were conducted to investigate the associations between knowledge levels and demographic factors. A *p*-value below 0.05 was considered statistically significant.

## Results

Based on the responses received, the respondents were then ordinally categorized into 4 knowledge groups, giving the % of total sample: 0 (none, 48%), 1–7 (poor, 26%), 8–11 (medium, 18%), 12–17 (good, 8%).

**Figure 1:**
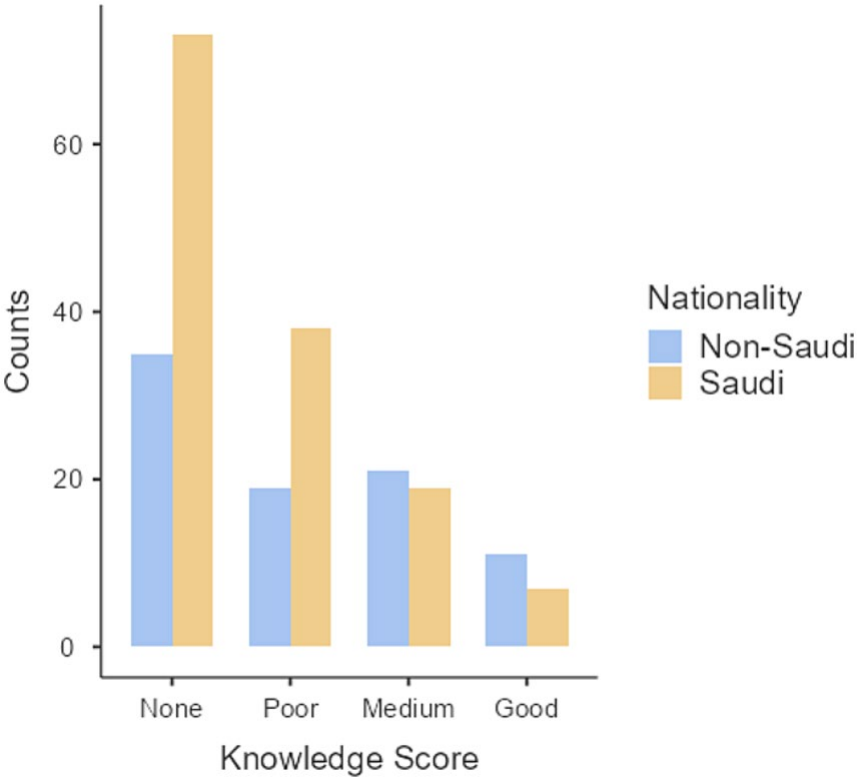
Distribution of Saudi and non-Saudi students across thalassemia knowledge categories: none, poor, medium, and good.

The resulting knowledge score categories were then entered as the dependent variable in an ordinal regression analysis. Age, gender, nationality, and marital status acted as predictor variables in the analysis. Age was a continuous covariate. The reference level for gender was female, for nationality it was Saudi, and for marital status it was single. McFadden’s R^2^ was 0.018. The following estimates (log odds ratios) and odds ratios were obtained from the analysis:

With all variables entered, nationality was the only variable to show a statistical effect, *p* < 0.01, as shown in Table 1. The odds ratio for this variable was 1.975, which, if other variables are held constant, indicates that non-Saudi nationals were almost twice as likely as Saudi nationals to be in a better knowledge score category. Descriptive comparisons by age and gender are presented in Table 2. A categorical analysis, not taking into account other variables, discerned a statistical trend across the ordinal knowledge score categories, Mantel–Haenszel χ^2^(1) = 8.07, *p* = 0.004. This trend in decreasing proportions of Saudis to non-Saudis, moving from knowledge score categories of None to Good, is illustrated in Figure 1.

**Table 1:** Ordinal regression analysis of predictors associated with thalassemia knowledge levels among non-medical university students.

Predictor	Estimate	SE	*z*	*p*	Odds ratio
Age	0.046	0.039	1.178	0.239	1.047
Gender: Male–female	−0.426	0.318	−1.34	0.18	0.653
Nationality: Non-Saudi–Saudi	0.681	0.263	2.592	0.01	1.975
Marital status: Married–single	−0.476	0.73	−0.652	0.514	0.621

**Table 2:** Comparison of demographic variables (age, gender, and nationality) among survey participants.

Characteristics and colleges of participants		Age in years		*p*-value (test statistic)
	N	Mean	SD	
Non-Saudi	86	21.6	5.2	*p* = 0.033
Saudi	137	20.3	2.8	(*t*(117) = 2.152)
Female	170	20.3	2.4	*p* = 0.045
Male	53	22.3	6.8	(*t*(56) = 2.052)
College of Business–Graduate (Masters or PhD)	2			
College of Business–Undergraduate	54			
College of Engineering–Graduate (Masters or PhD)	6			
College of Engineering–Undergraduate	101			
College of Law–Undergraduate	16			
College of Pharmacy–Graduate (Masters or PhD)	2			
College of Pharmacy–Undergraduate	5			
College of Science–Graduate (Masters or PhD)	18			
College of Science–Undergraduate	19			

	Nationality			
	Non-Saudi	Saudi	Total	
Female	63	107	170	*p* = 0.410
Male	23	30	53	(*G*(1) = 0.679)
Total	86	137	223	

## Discussion

### Comparison with existing literature

#### Knowledge levels

The results of this study are consistent with previous studies in high-prevalence countries. Nearly half (48%) of participants in this study received a zero on the thalassemia knowledge evaluation, with just 8% achieving a satisfactory knowledge level. The findings are consistent with those of (12), who found that 48% of students at King Abdulaziz University had ever heard of thalassemia, and the average knowledge score was poor, with particular concern that 50% of the married students had never heard of the disease, despite mandated premarital screening. Similarly, (6) in Malaysia, it was discovered that while 68.6% of participants had heard of thalassemia, the majority could not accurately identify it or distinguish between carriers and affected individuals.

In our study, 69.5% of university students had never heard of thalassemia, and nearly half (48%) got a zero on the knowledge assessment. Moreover, among individuals familiar with the disease, significant misconceptions persisted—only 39.9% accurately identified thalassemia as a genetic disorder, and many respondents were uncertain or incorrect about its transmissibility, symptoms, and modes of inheritance. These findings indicate elevated levels of misinformation and a deficient baseline knowledge, which matches research conducted among high-risk populations. For example, (7) reported that only 44.6% of parents of thalassemia patients in Pakistan recognized the hereditary basis of the disorder, suggesting that even those immediately affected by the disease might have a fundamental lack of understanding of its characteristics.

The ordinal regression analysis indicated that nationality was the only statistically significant predictor of thalassemia knowledge (*p* < 0.01), with non-Saudi students exhibiting nearly double the knowledge scores (OR = 1.975). This might represent disparities in public health awareness or school curriculum across countries. While (12) found that students who studied outside of Saudi Arabia had poorer knowledge, potentially due to less local exposure to the national premarital screening program, our data indicate that non-Saudis in our sample had higher levels of awareness. Our findings are consistent with (6), who found that awareness differed considerably by cultural and ethnic background. Similarly, (5) found that Bangladeshi college students from urban regions and scientific disciplines had much greater knowledge than their counterparts from the rural areas or arts-related fields. This shows that the educational setting and environment, which might vary depending on nationality, can play an essential role in shaping awareness.

The study reveals that non-Saudi students have significantly better knowledge of thalassemia than Saudi students, potentially due to differences in their secondary school curricula that emphasize genetic diseases more thoroughly. Other factors may include their prior exposure to genetic counseling and public health initiatives in their home countries, which could enhance their understanding of premarital screening and carrier status. However, these observations are a venue for discussion and reflection, as the study did not assess previous educational backgrounds or sources of health information.

#### Public health significance

The study’s findings of poor knowledge and widespread misunderstandings imply that Saudi Arabia’s required premarital screening program is not supported by adequate public health education (12). Individuals who do not comprehend the goal or implications of screening might undergo testing passively, without making informed reproductive decisions. This outcome is concerning as several students believed that thalassemia was either communicable or not genetically related, thereby perpetuating stigma and anxiety among carriers and patients (5). These findings highlight a critical gap in health literacy, limiting the preventative efficacy of current screening initiatives.

#### Educational needs

The study’s findings highlight the importance of developing structured educational interventions to overcome the considerable gaps in thalassemia knowledge among university students and even middle-high and high school students. Similar initiatives in other settings have resulted in meaningful increases in public awareness and screening behaviors. For example, video-based sessions for Indonesian high school students boosted information retention (13), whereas classroom programs in Turkey dramatically raised middle school students’ awareness (9). Community-based education in Cambodia increased both knowledge and desire to get screened (10). Notably, an intervention in Indonesia involving thalassemia patients and their families as educators resulted in enhanced understanding and increased motivation for genetic testing (4). These findings support creating culturally relevant university-level educational campaigns that engage students using interactive, peer-led, and media-supported modalities.

#### Strengths and limitations of the study

Some limitations in this study should be acknowledged. First, convenience sampling from a single private university in Riyadh introduces selection bias. It restricts the implications of the results to the larger population of Saudi university students, particularly those from public institutions or from other parts of the country. Second, the survey’s self-administered online format increases the likelihood of self-report bias, including social desirability effects and random guessing, especially among individuals unfamiliar with thalassemia. Third, future studies should include variables such as participants’ sources of health information, previous genetic education exposure, and family history of thalassemia to clarify factors affecting knowledge levels, as these aspects were not assessed in the study. Despite these limitations, the study offers valuable preliminary insights regarding awareness gaps and educational needs among a young, educated population.

## Conclusion

This study investigated the awareness and knowledge of thalassemia among non-medical college students at Alfaisal University. Our findings demonstrated a prevalent lack of understanding about thalassemia. Nearly half of the participants (48%) got zero on the knowledge evaluation, with only 8% showing good understanding. Moreover, 69.5% reported that they had never heard of thalassemia, highlighting a significant awareness gap. When evaluating factors associated with knowledge levels, ordinal regression analysis revealed that nationality was the sole significant predictor, with non-Saudi students approximately twice as likely as Saudis to fall into the higher knowledge category. No significant associations were discovered for age, gender, or marital status.

### Recommendations and future research

According to the findings of this study, there is a definite need for educational initiatives aimed at non-medical university students to improve knowledge of thalassemia and dispel widespread misunderstandings. The findings imply a lack of understanding at the community level before the students start university. Community education is an essential part of thalassemia control programs and, unlike education given to susceptible families for genetic counseling and prenatal diagnosis, it should cover the entire target population.

Different segments of society should be informed through education in designated groups such as students, teachers, community leaders, soldiers, and nongovernmental organizations to reach as wide a part of society as possible. Priority should be given to providing training in the target audiences’ locations. So, schools, military units, workplaces, villages, events led by nongovernmental organizations, etc., can be counted among these. Larger events that will include wider segments of society can also be organized. The primary institution responsible for health education at the national level is the Ministry of Health, and its support must be received for public education programs.

Additionally, it is important to receive support and contributions from the Ministry of Education and universities for educational activities in schools and universities. The Red Crescent and non-governmental organizations are highly encouraged to be involved in the public education programs. Universities could also consider incorporating genetic literacy into general health education programs or offering elective workshops by healthcare professionals and patient advocates.

Future studies should explore the efficacy of such programs by administering pre- and post-education tests to determine changes in knowledge, attitudes, and screening behavior. Furthermore, expanding this study to include students from public institutions, different regions of Saudi Arabia, and other age groups will offer a more complete picture of awareness levels in the general community. Longitudinal studies can also assist in determining if increasing knowledge leads to substantial behavioral changes, such as informed premarital decision-making or higher screening uptake.

## Data Availability

The raw data supporting the conclusions of this article will be made available by the authors, without undue reservation.

## Footnotes

1
https://www.jamovi.org

## References

[1] Aljabry M Sulimani S Alotaibi G Aljabri H Alomary S Aljabri O . Prevalence and regional distribution of beta-hemoglobin variants in Saudi Arabia: insights from the national premarital screening program. J Epidemiol Glob Health. (2024) 14:1242–8. doi: 10.1007/s44197-024-00281-x, 39073533 PMC11442792

[2] Alsaeed ES Farhat GN Assiri AM Memish Z Ahmed EM Saeedi MY . Distribution of hemoglobinopathy disorders in Saudi Arabia based on data from the premarital screening and genetic counseling program, 2011–2015. J Epidemiol Glob Health. (2018) 7 Suppl 1:S41–7. doi: 10.1016/j.jegh.2017.12.001, 29801592 PMC7386442

[3] Armeli C Robbins SJ Eunpu D. Comparing knowledge of β-thalassemia in samples of Italians, Italian-Americans, and non-Italian-Americans. J Genet Couns. (2005) 14:365–76. doi: 10.1007/s10897-005-1123-5 16195943

[4] Asa P Indiastuti DN Andarsini MR Fauziah JN d’Arqom A. Empowering thalassemia patients and family to increase public knowledge on thalassemia. J Pengabdi Kpd Masy Indones J Community Engagem. (2021) 7:228–33. doi: 10.22146/jpkm.69349

[5] Hossain MS Hasan MM Raheem E Islam MS Al Mosabbir A Petrou M . Lack of knowledge and misperceptions about thalassaemia among college students in Bangladesh: a cross-sectional baseline study. Orphanet J Rare Dis. (2020) 15:54. doi: 10.1186/s13023-020-1323-y 32085790 PMC7035777

[6] Wong LP George E Tan JAMA. A holistic approach to education programs in thalassemia for a multi-ethnic population: consideration of perspectives, attitudes, and perceived needs. J Community Genet. (2011) 2:71–9. doi: 10.1007/s12687-011-0039-z, 22109791 PMC3186023

[7] Ishaq F Abid H Kokab F Akhtar A Mahmood S. Awareness among parents of β-thalassemia major patients regarding prenatal diagnosis and premarital screening. J Coll Physicians Surg Pak. (2012) 22:218–21. 22482376

[8] Rakhmilla L. Assessing knowledge about thalassemia among reproductive age population after video media education. (2018). Available online at: https://www.academia.edu/80779918/Assessing_Knowledge_About_Thalassemia_Among_Reproductive_Age_Population_After_Video_Media_Education (Accessed May 11, 2025).

[9] Arpacı A Aytaç N Yüregir GT Tuli A Aksoy K. An education programme on sickle cell anemia and β-thalassemia for the 8th grade students. Turk J Haematol. (2003) 20:19–24. 27265330

[10] Cheng K Fucharoen S Sanchaisuriya K Fucharoen G Sanchaisuriya P Jetsrisuparb A. Effect of health education on severe thalassemia prevention and control in communities in Cambodia. Arch Public Health. (2018) 76:13. doi: 10.1186/s13690-018-0259-3 29479428 PMC5817790

[11] Tutuba HJ Jonathan A Lloyd W Masamu U Marco E Makani J . The efficacy of maternal health education and maternal screening on knowledge and the uptake of infant screening for sickle cell disease in Dar-Es-Salaam, Tanzania: a quasi-experimental study. BMC Public Health. (2023) 23:70. doi: 10.1186/s12889-022-14859-2 36627609 PMC9832626

[12] Olwi DI Merdad LA Ramadan EK. Thalassemia: a prevalent disease yet unknown term among college students in Saudi Arabia. J Community Genet. (2018) 9:277–82. doi: 10.1007/s12687-017-0351-3, 29238908 PMC6002305

[13] Rakhmilla LE Larasati R Sahiratmadja EK Rohmawaty E Susanah S Effendi SH. Assessing knowledge about thalassemia among reproductive age population after video media education. J Biomed Clin Sci. (2018) 2:30–2.

[14] Almasmoum HA Tabassum A Iqbal MS Abo-Alshamat R Aqeeli W. Knowledge and attitude toward hemoglobinopathies in premarital screening program among the general population in the western region of Saudi Arabia. Hemoglobin. (2022) 46:277–84. doi: 10.1080/03630269.2022.2142607, 36369918

[15] Al Sulaiman A Suliman A Al Mishari M Al Sawadi A Owaidah TM. Knowledge and attitude toward the hemoglobinopathies premarital screening program in Saudi Arabia: a population-based survey. Hemoglobin. (2008) 32:531–8. doi: 10.1080/03630260802508384, 19065330

[16] Eissa M Patel AA Farag S Babiker NH Al-Shahrani MS Al-Nahari AM . Awareness and attitude of university students about screening and testing for hemoglobinopathies: a case study of the Aseer region, Saudi Arabia. Hemoglobin. (2018) 42:264–8. doi: 10.1080/03630269.2018.1541802, 30821195

